# Size-selected Fe_3_O_4_–Au hybrid nanoparticles for improved magnetism-based theranostics

**DOI:** 10.3762/bjnano.9.251

**Published:** 2018-10-16

**Authors:** Maria V Efremova, Yulia A Nalench, Eirini Myrovali, Anastasiia S Garanina, Ivan S Grebennikov, Polina K Gifer, Maxim A Abakumov, Marina Spasova, Makis Angelakeris, Alexander G Savchenko, Michael Farle, Natalia L Klyachko, Alexander G Majouga, Ulf Wiedwald

**Affiliations:** 1 Department of Chemistry, Lomonosov Moscow State University, Moscow, 119991, Russia; 2 National University of Science and Technology «MISIS», Moscow, 119049, Russia; 3 Physics Department, Aristotle University of Thessaloniki, Thessaloniki, 54124, Greece; 4 Department of Medical Nanobiotechnology, Russian National Research Medical University, Moscow, 117997, Russia; 5 Faculty of Physics and Center for Nanointegration Duisburg-Essen, University of Duisburg-Essen (CENIDE), Duisburg, 47057, Germany; 6 D. Mendeleev University of Chemical Technology of Russia, Moscow, 125047, Russia

**Keywords:** hybrid nanoparticles, magnetic hyperthermia, magnetic resonance imaging, nanomagnetism, theranostics

## Abstract

Size-selected Fe_3_O_4_–Au hybrid nanoparticles with diameters of 6–44 nm (Fe_3_O_4_) and 3–11 nm (Au) were prepared by high temperature, wet chemical synthesis. High-quality Fe_3_O_4_ nanocrystals with bulk-like magnetic behavior were obtained as confirmed by the presence of the Verwey transition. The 25 nm diameter Fe_3_O_4_–Au hybrid nanomaterial sample (in aqueous and agarose phantom systems) showed the best characteristics for application as contrast agents in magnetic resonance imaging and for local heating using magnetic particle hyperthermia. Due to the octahedral shape and the large saturation magnetization of the magnetite particles, we obtained an extraordinarily high *r*_2_-relaxivity of 495 mM^−1^·s^−1^ along with a specific loss power of 617 W·g_Fe_^−1^ and 327 W·g_Fe_^−1^ for hyperthermia in aqueous and agarose systems, respectively. The functional in vitro hyperthermia test for the 4T1 mouse breast cancer cell line demonstrated 80% and 100% cell death for immediate exposure and after precultivation of the cells for 6 h with 25 nm Fe_3_O_4_–Au hybrid nanomaterials, respectively. This confirms that the improved magnetic properties of the bifunctional particles present a next step in magnetic-particle-based theranostics.

## Introduction

Biocompatible magnetite nanoparticles (NPs) are anticipated to provide new noninvasive therapies and early diagnostics for previously incurable diseases using a single, so-called “theranostics” platform [1–3]. The magnetic properties of Fe_3_O_4_ NPs give rise to novel therapeutic approaches such as magneto-mechanical cancer treatment [4] and magnetic particle hyperthermia (MPH) [5–7] as well as to improvements in diagnostic techniques like magnetic resonance imaging (MRI) [8–10] and magnetic particle imaging (MPI) [11–12]. For advanced functionality under real operational conditions, various approaches have been attempted, e.g., optimization of the NP surface using targeting molecules or specific polymers [13–15]. From the physics point of view, the strategically tailored design of structural and magnetic properties in biocompatible Fe_3_O_4_ NPs is of utmost importance for improved performance in MPH, MRI, or MPI [16–17]. It is essential to obtain Fe_3_O_4_ NPs of high crystallinity with bulk-like magnetic properties, which change with the NP size, shape and iron oxidation state [18–20]. These parameters can be adjusted by heterogeneous nucleation of NPs on noble metal seeds [21–22]. Additionally, such bifunctional Fe_3_O_4_–Au NPs are potentially applicable for targeted drug delivery, enhanced hyperthermia, multimodal imaging and theranostics [8,23–27].

In this work, we present the first size-dependent study of hybrid Fe_3_O_4_–Au NPs with Janus structure for application in theranostics where improvements in MRI and MPH were demonstrated. Increasing the magnetic NP diameter from 6 to 44 nm, we show the gradual transition of their lattice parameters from an intermediate value between maghemite γ-Fe_2_O_3_ and magnetite, to high-quality stoichiometric Fe_3_O_4_. We find a size-dependent transition from superparamagnetic to a stable ferrimagnetic response, a bulk-like saturation magnetization, and observe the Verwey transition at 123 K – all of which result in the superior magnetic properties for a particle diameter greater than 20–25 nm [28].

For theranostic application, we test the contrast enhancement of the developed materials in MRI and the heating potential in MPH. Importantly, these measurements are performed in both aqueous and agarose dispersions, i.e., phantoms, mimicking the conditions in cells and tissues. For the MRI tests, we observe the growth of the *r*_2_-relaxivity from 159 to 495 mM^−1^·s^−1^ in water and from 118 to 612 mM^−1^·s^−1^ in agarose gel matrices with increasing NP diameter from 6 to 25 nm. Our best values are significantly enhanced in comparison to other Fe_3_O_4_–Au hybrids or commercial contrast agents due to the high crystallinity and large bulk-like saturation magnetization leading to larger field gradients in MRI. The MPH measurements reveal that the specific loss power (SLP) increases from 10 to 617 W·g_Fe_^−1^ in water and from 12 to 327 W·g_Fe_^−1^ in agarose with increasing NP diameter from 6 to 25 nm. The 25 nm and 44 nm diameter NPs show similar theranostic performance.

In in vitro experiments we detected the death of 4T1 mouse breast cancer cells at a rate of 79 ± 8% after exposure to 25 nm Fe_3_O_4_–Au hybrids for 30 min in an ac magnetic field (AMF) with 261–393 kHz and 25 mT, which resulted in heating up to 46 ± 1 °C. Preincubation of the cells with the hybrid NPs for 6 h further decreased the cell viability and led to complete (100%) cell death. Such multifunctional Fe_3_O_4_–Au Janus NPs combine the best characteristics for MRI and MPH and offer the highest potential for therapeutic and visualization capabilities in magnetism-based theranostics.

## Results and Discussion

In this section, we start presenting the basic characterization of the size-selected NPs addressing dimensions and morphology, structure, and magnetic properties. This is followed by a discussion of the theranostic application of NPs in MRI and MPH. We conclude with a proof-of-principle in vitro study showing efficient induction of cell death.

### Size and morphology

All Fe_3_O_4_–Au hybrid NPs were synthesized by the thermal decomposition of iron pentacarbonyl on the surface of Au NPs in a high-boiling solvent. Details regarding the synthesis are given in the Experimental section. In brief, Fe_3_O_4_ was grown on either in situ synthesized Au NPs (samples MNP-6 and MNP-15) or presynthesized Au seeds (samples MNP-25 and MNP-44). In addition, by using three different solvents (phenyl ether, benzyl ether, 1-octadecene), we vary the reaction temperature. The sample numbers reflect the mean magnetic NP diameter, i.e., the Fe_3_O_4_ part, in nanometers. After synthesis, all NPs were investigated by transmission electron microscopy (TEM).

**Figure 1:**
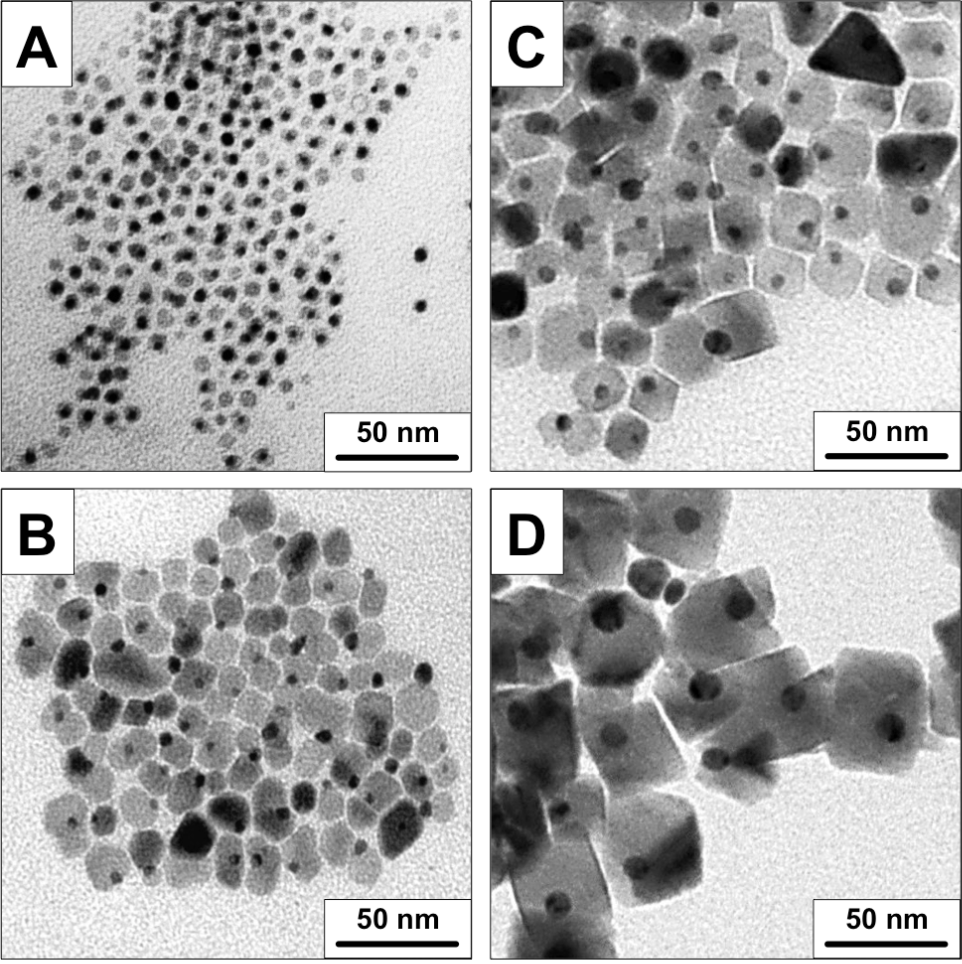
Bright-field TEM images of size-selected magnetite–gold NPs with in situ synthesized Au seeds: A) MNP-6; B) MNP-15; and with presynthesized Au seeds: C) MNP-25; D) MNP-44. The sample numbers reflect the Fe_3_O_4_ NP mean size in nanometers.

Figure 1 shows the corresponding images of the four NP batches: magnetite and gold NPs are pairwise connected and form hybrid NPs. The magnetite NPs formed using the in situ synthesized Au seeds have a spherical or poorly facetted shape (Figure 1A and 1B), while NPs obtained using presynthesized Au seeds are highly facetted (Figure 1C and 1D). The formation of highly facetted magnetite with improved crystallinity in this case is likely due to the longer reflux time. To the best of our knowledge, only a few examples of Fe_3_O_4_–Au NPs with octahedral-like morphology have been reported in the literature [29–30]. We find that our hybrid NPs outperform previous reports in terms of size selection, size distribution, degree of crystallinity and faceting.

The size distribution of the magnetite–gold NPs was measured based on a series of TEM images. The average NP diameter and standard deviation (SD) values are presented in Table 1. The size histograms are provided in Supporting Information File 1, Figure S1. The volume fraction of magnetite and gold can be evaluated using the average NP diameter (assuming spherical Au–Fe_3_O_4_ NPs) by TEM and from fitting by modified Rietveld refinement from XRD. Importantly, the ratio of Fe_3_O_4_ to Au is almost constant at about 97% for MNP-15, MNP-25, and MNP-44, while for the smallest NPs, we obtain a slightly lower Fe_3_O_4_ volume fraction of 91%. For optimized MRI and MPH properties, the Fe_3_O_4_ volume fraction should be high since the diamagnetic Au can only be considered as a minor contributor. In comparison to Fe_3_O_4_, Au does not modify the optimized collective magnetic response, which is a prerequisite for biomagnetic applications.

**Table 1:** Results of the structural and morphological characterization by TEM and XRD. The NP size distribution, volume fraction of Fe_3_O_4_ and Au, lattice parameter (*a*), and crystallite size are listed.

Sample	TEM				XRD					
	NP diameter (nm)		Volume fraction (%)		*a* (nm)		Crystallite diameter (nm)		Volume fraction (%)	
	Fe_3_O_4_	Au	Fe_3_O_4_	Au	Fe_3_O_4_	Au	Fe_3_O_4_	Au	Fe_3_O_4_	Au

MNP-6	6.3 ± 0.8	3.2 ± 0.6	91.4 ± 4.1	8.6 ± 4.1	0.8376 ± 0.0005	0.4060 ± 0.0005	4.0 ± 1.0	2.0 ± 1.5	92.9 ± 4.0	7.1 ± 4.0
MNP-15	14.6 ± 2.7	5.9 ± 1.0	96.9 ± 2.3	3.1 ± 2.3	0.8384 ± 0.0004	0.4068 ± 0.0004	15.0 ± 2.0	4.0 ± 1.0	95.5 ± 2.0	4.5 ± 2.0
MNP-25	25.1 ± 5.0	9.2 ± 2.1	97.2 ± 2.6	2.8 ± 2.6	0.8394 ± 0.0002	0.4076 ± 0.0002	26.0 ± 1.1	4.5 ± 0.4	95.3 ± 0.7	4.7 ± 0.7
MNP-44	43.9 ± 10.6	10.9 ± 2.3	97.1 ± 2.6	2.9 ± 2.6	0.8390 ± 0.0001	0.4082 ± 0.0002	16.8 ± 0.4	9.5 ± 0.6	95.0 ± 1.5	5.0 ± 1.5

### Structure and phase composition

**Figure 2:**
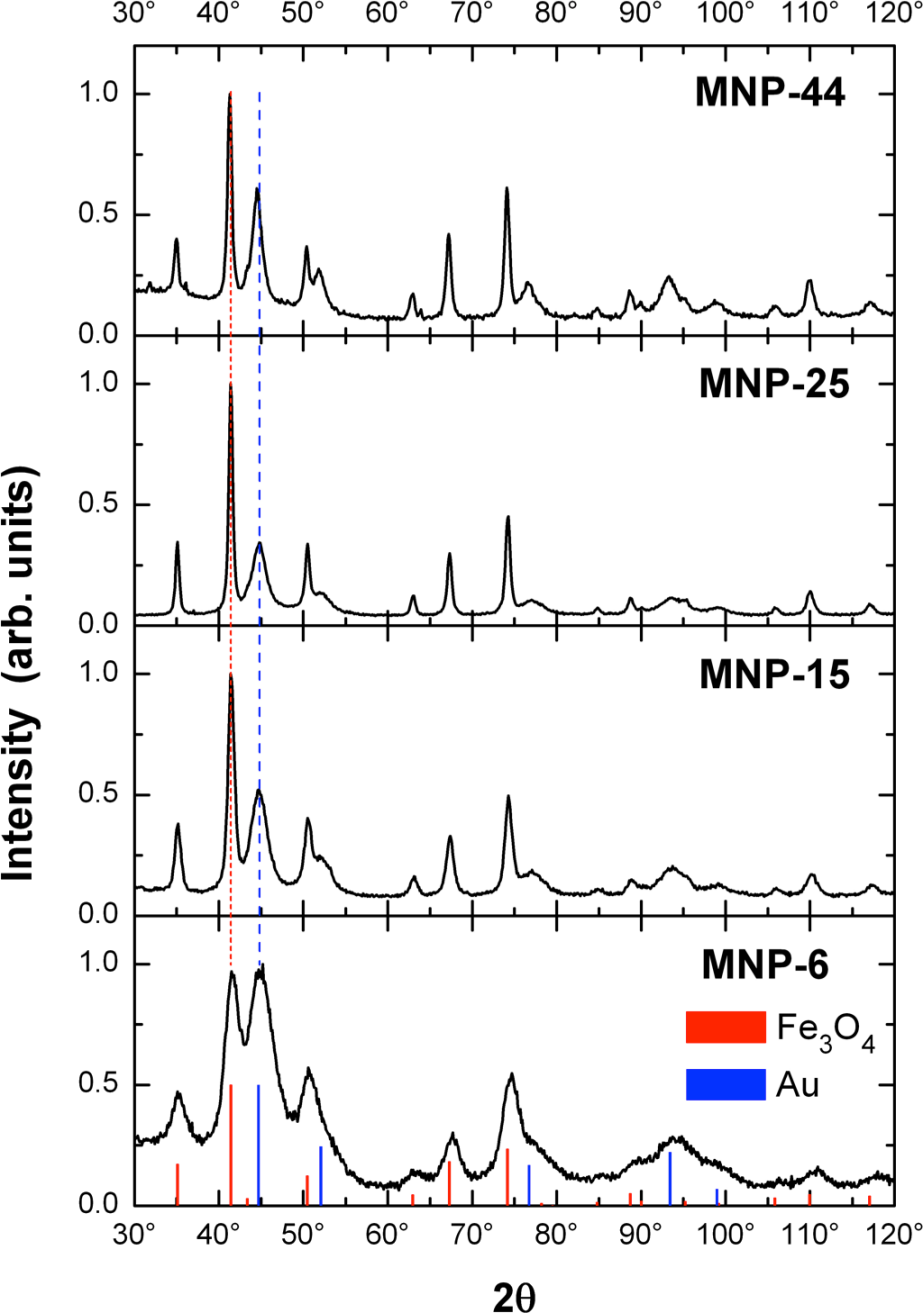
XRD patterns of Fe_3_O_4_–Au NPs. Panels are sorted by magnetic NP size from bottom to top – samples MNP-6, MNP-15, MNP-25 and MNP-44, respectively. The intensity of each diffractogram is normalized to the strongest peak. The red and blue vertical lines represent the angular position and relative intensity of reference bulk magnetite and gold phases.

The structure and phase composition of the Fe_3_O_4_–Au NPs was investigated by X-ray diffraction (XRD). Figure 2 presents the experimental data. All expected powder diffraction peaks of magnetite and gold are clearly observed. Rietveld refinement, combining the powder diffraction reference data of Fe_3_O_4_ (ICDD PDF-2 No. 00-019-0629) and Au (ICDD PDF-2 No. 03-065-8601), is applied (not shown). The extracted lattice constants, crystallite size and phase volume fractions are listed in Table 1.

Since magnetite (Fe_3_O_4_) and maghemite (γ-Fe_2_O_3_) are structurally similar, XRD alone does not provide an accurate discrimination between the two phases. As listed in Table 1, the lattice parameter approaches bulk Fe_3_O_4_ (*a* = 0.8397 nm) rather than bulk γ-Fe_2_O_3_ (*a* = 0.8347 nm) with increasing NP size [31]. The XRD results suggest that the structure of sample MNP-25 is bulk-like Fe_3_O_4_ and Au in the NPs. The TEM diameter and the XRD crystallite size measurements of Fe_3_O_4_ match well, except for the MNP-44 batch, where polycrystalline Fe_3_O_4_ is presumed. We conclude that the Fe_3_O_4_ crystallite size can be varied while holding the Fe_3_O_4_/Au phase volume ratio almost constant.

**Figure 3:**
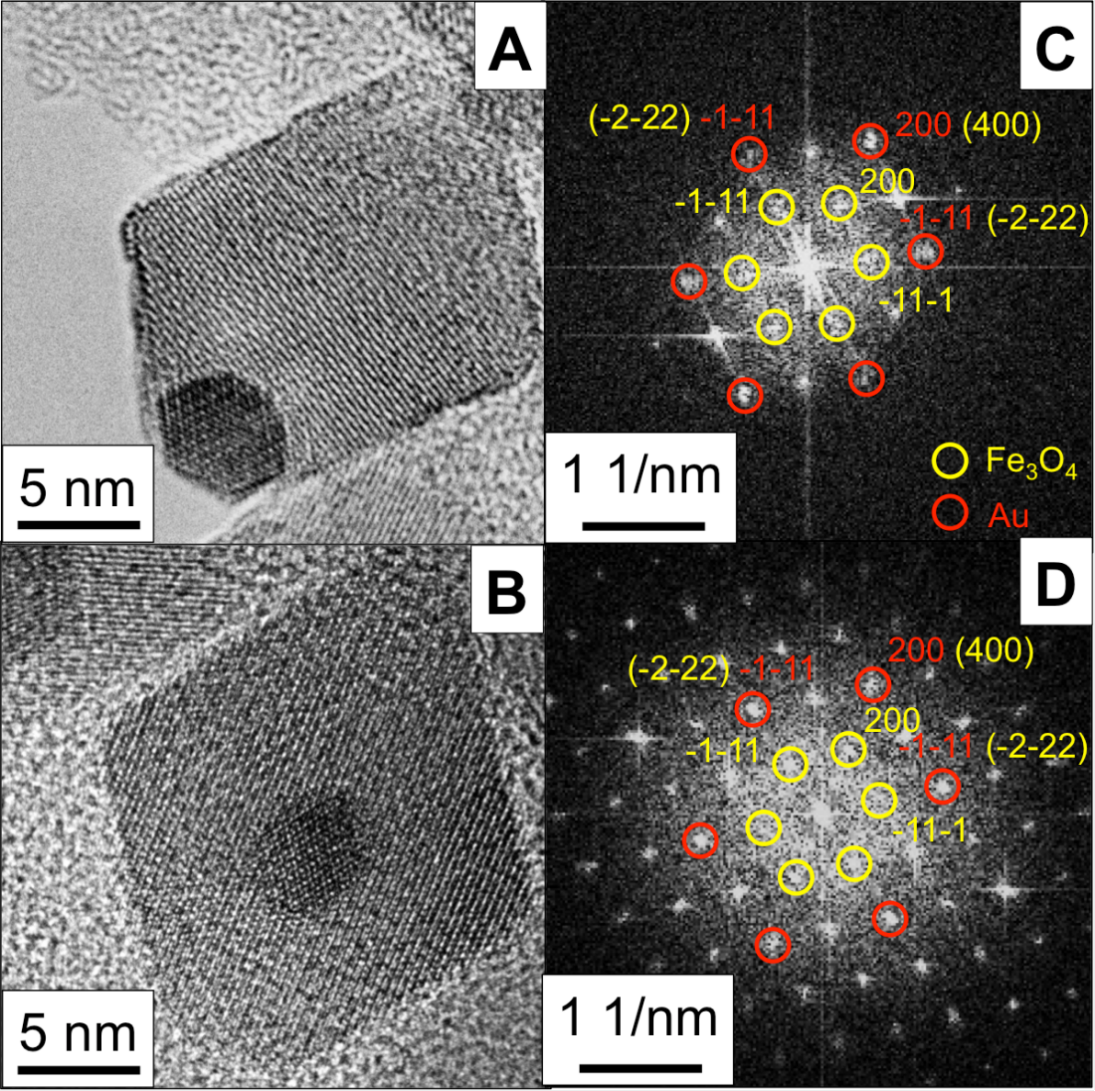
HRTEM and corresponding FTT images of size-selected magnetite–gold NPs: MNP-15 (A, C) and MNP-25 (B, D). Fe_3_O_4_ and Au indices are marked yellow and red, respectively. The [111] and [200] crystallographic directions of Fe_3_O_4_ and Au register to each other. The NPs are viewed along their [011] direction.

Additionally, the crystallographic orientation of Fe_3_O_4_ and Au for samples MNP-15 (with in situ synthesized Au seeds) and MNP-25 (with presynthesized Au seeds) was evaluated using bright-field high-resolution TEM (HRTEM) imaging (Figure 3A and 3B) and fast Fourier transform (FFT) (Figure 3C and 3D). It is clear that the Au NPs, acting as seeds in the synthesis, allow for epitaxial growth of Fe_3_O_4_ on Au, forming the Janus structure with Au (111) || Fe_3_O_4_ (111) and Au (200) || Fe_3_O_4_ (200), which is in agreement with the previous reports on similar hybrids and electrodeposited epitaxial films [32–33]. HRTEM images of samples MNP-6 and MNP-44 are presented in Supporting Information File 1, Figure S2. While MNP-44 shows a similar growth mode, the smallest hybrid NPs (MNP-6) show a rather spherical shape for the Au core and deteriorated Fe oxide parts attached to each other.

The composition (in terms of Fe_3_O_4_ and Au mass fraction) of the hybrid NPs is determined by XRD (assuming Fe_3_O_4_ and Au bulk densities of 5.2 g·cm^−3^ and 19.3 g·cm^−3^, respectively) and additionally by atomic emission spectrometry (AES). The results are presented in Table 2. XRD and AES data for the samples MNP-15, MNP-25 and MNP-44 correlate well within 3%. The larger difference for MNP-6 can be explained by the presumably larger fraction of maghemite in this sample. For all further analysis we use the AES results.

**Table 2:** Mass fraction of Fe_3_O_4_ and Au in the samples, as determined by XRD and AES analysis.

Sample	mass % (XRD)		mass % (AES)	
	Fe_3_O_4_	Au	Fe_3_O_4_	Au

MNP-6	78.0 ± 4.0	22.0 ± 4.0	84.7 ± 2.3	15.3 ± 2.3
MNP-15	85.2 ± 2.0	14.8 ± 2.0	82.9 ± 1.4	17.1 ± 1.4
MNP-25	84.6 ± 0.7	15.4 ± 0.7	85.3 ± 0.9	14.7 ± 0.9
MNP-44	83.8 ± 1.5	16.2 ± 1.5	87.5 ± 2.5	12.5 ± 2.5

### Magnetic properties

**Figure 4:**
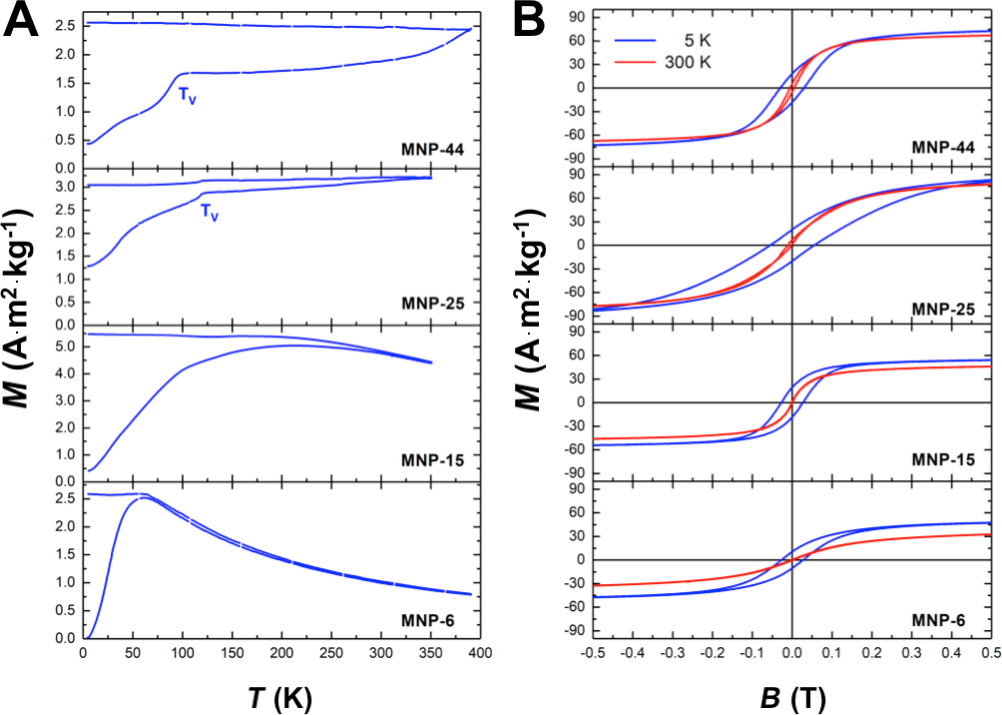
Magnetic properties of Fe_3_O_4_–Au hybrid NPs. ZFC/FC curves at *B* = 5 mT (A). *T*_V_ indicates the Verwey transition temperature for samples MNP-25 and MNP-44. Hysteresis loops at *T* = 5 K and *T* = 300 K (B).

The static magnetic properties are presented in this section. We measure zero-field cooling and field cooling (ZFC/FC) at an applied field of µ_0_*H* = 5 mT and hysteresis loops in the field range µ_0_*H* = ±9 T in the temperature interval 5–350 K for MNP-15 and MNP-25 and 5–390 K for samples MNP-6 and MNP-44. Figure 4A presents the ZFC/FC curves. With increasing NP size, the superparamagnetic blocking temperature (*T*_B_) increases from 62 K for MNP-6 to 210 K for MNP-15, as identified by the maximum of the ZFC branch. However, the rather broad size distribution of 10–20% (Table 1) and corresponding volume distributions result in broad distributions of blocking temperatures, *T*_B_. Thus, the *T*_B_ values should be taken as those of the larger NPs. For sample MNP-25, *T*_B_ is above ambient and the ZFC curve suggests a *T*_B_ in the range 310–350 K while the *T*_B_ of sample MNP-44 is clearly larger than the experimentally accessible temperature range 5–390 K. For samples MNP-25 and MNP-44, a sudden increase of the ZFC curve is observed above 123 K and 100 K, respectively, after which a plateau develops. We identify this feature as the Verwey transition in Fe_3_O_4_ at *T*_V_ = 123 K in bulk material [34]. Sample MNP-25 shows the bulk *T*_V_, revealing the high quality of the Fe_3_O_4_ nanocrystals. Instances of slightly off-stoichiometric Fe_3_O_4_ leads to rather large shifts of *T*_V_ towards lower temperatures [35]. Therefore, the Verwey transition could often not be identified in NP ensembles since the transition smears out over a broader temperature range [36]. For sample MNP-44, *T*_V_ is indeed shifted by more than 20 K, indicating that the Fe_3_O_4_ in this sample is of lower quality than for sample MNP-25. We ascribe this to the polycrystalline nature of the Fe_3_O_4_ NPs for this sample, as supported by the XRD results revealing a grain size of 17 nm compared to the TEM diameter of 44 nm. In contrast, the diameter of Fe_3_O_4_ in sample MNP-25, as determined by TEM and XRD, is the same within the error bar.

Figure 4B shows the magnetic hysteresis loops at *T* = 5 K and *T* = 300 K. All samples have an open hysteresis at *T* = 5 K while at ambient temperature only the larger NPs (MNP-25 and MNP-44) preserve their hysteretic behavior. Smaller NPs become superparamagnetic in accordance with the ZFC/FC curves in Figure 4A. Note that for Fe_3_O_4_ the transition from a single- to multidomain state is expected at a critical diameter of 30–90 nm, depending on the magneto-crystalline anisotropy and the saturation magnetization *M*_S_ as well as on the shape and morphology [37–39]. We thus expect that except for sample MNP-44 all magnetite hybrid samples are single domain.

The *M*_S_ (Table 3) is measured at large fields by extrapolation of a linear fit to the ordinate. The *M*_S_ increases with increasing particle size from 57.0 (47.6) A·m^2^·kg^−1^ (Fe_3_O_4_) for MNP-6 to 97.1 (86.8) A·m^2^·kg^−1^ (Fe_3_O_4_) at 5 (300) K for MNP-25 while MNP-44 only reaches 79.6 (73.6) A·m^2^·kg^−1^ (Fe_3_O_4_) at 5 (300) K probably due to the reduced grain size and resulting deterioration of the Fe_3_O_4_ lattice as well as partial oxidation to γ-Fe_2_O_3_, e.g., at the grain boundaries. The decrease of *M*_S_ for small particles has been ascribed to these features [40–42] and considering the bulk *M*_S_ values at 5 K (96.4 A·m^2^·kg^−1^ for magnetite) and 300 K (92.0 A·m^2^·kg^−1^ for magnetite and 76.0 A·m^2^·kg^−1^ for maghemite) our results follow the trends reported previously [43–46]. Note that *M*_S_ of sample MNP-25 matches the Fe_3_O_4_ bulk value within the error bar. The error is rather large due to the net weight of the samples (few milligrams) and the mass fraction of Fe oxide with respect to Au as determined by AES (Table 2).

**Table 3:** Overview of the size-dependent magnetic properties of Fe_3_O_4_–Au NPs. Saturation magnetization *M*_S_ at 9 T, *T* = 5 K and *T* = 300 K, coercive field µ_0_*H*_C_ at *T* = 5 K, and deduced blocking temperature, *T*_B,_ and effective magnetic anisotropy, *K*_eff_. The bulk Fe_3_O_4_ reference values are listed for comparison and referenced in the text.

Sample	*M*_S_ (A·m^2^·kg(Fe_3_O_4_)^−1^)		µ_0_*H*_C_ (mT)	*T*_B_ (K)	*K*_eff_ (kJ·m^−3^)
	*T* = 5 K	*T* = 300 K	*T* = 5 K	ZFC	Sharrock model

MNP-6	57.0 ± 3.0	47.6 ± 2.4	27 ± 2	62	45 ± 18
MNP-15	70.4 ± 2.1	61.1 ±2.0	28 ± 2	210	11 ± 7
MNP-25	97.1 ± 2.4	86.8 ±2.1	55 ± 2	310	10 ± 6
MNP-44	79.6 ± 4.6	73.6 ± 4.2	30 ± 2	>390	–
bulk Fe_3_O_4_	96.4	92.0	–	–	13 (*K*_1_)

The temperature dependence of the coercive field *H*_C_(*T*) allows us to estimate the effective magnetic anisotropy energy density *K*_eff_ (Table 3) by using Sharrock’s equation for single domain, randomly oriented, non-interacting NPs [47–49]:


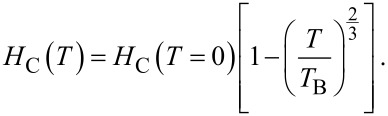


Random orientation and single domain properties are guaranteed for the three smaller particle batches [37], while dipolar coupling in the powder sample is neglected in the following. This simple approach averages over all particle sizes of a distribution and does not overestimate the mean blocking temperature as the ZFC curves do for larger size distributions [50]. Fitting *H*_C_(*T*) yields an average blocking temperature *T*_B_ which can be translated into *K*_eff_ via 21·*k*_B_*T* ≈ *K*_eff_·*V* where *k*_B_ the Boltzmann constant and *V* the NP volume. The prefactor 21 accounts for an attempt frequency of 10^9^ Hz and the VSM measurement time of 1 s [51].

Table 3 lists the extracted *K*_eff_ values while the uncertainty intervals have been estimated based on the volume distributions. We obtain 11 ± 7 kJ·m^−3^ and 10 ± 6 kJ·m^−3^ for MNP-15 and MNP-25, respectively. These values are in reasonable agreement with the first order anisotropy constant of bulk Fe_3_O_4_ *K*_1_ = 13 kJ·m^−3^ [37]. For MNP-44 the model is not used since *T*_B_ is much larger than the accessible temperature range and presumably rather close to the temperature where this simple model cannot be applied. More interesting is the significantly enhanced *K*_eff_ = 45 ± 18 kJ·m^−3^ for MNP-6. Previous reports and our XRD results suggest that small particles (<10–15 nm) crystallize as a composite of magnetite and maghemite, and the anisotropy constant increases with decreasing size. Martinez et al. [52], for example, determined *K*_1_ ≈ 70 kJ·m^−3^ for about 15 nm maghemite NPs, which is strongly enhanced as compared to the bulk value of *K*_1_ ≈ 4–5 kJ·m^−3^ [53]. The effective anisotropy of *K*_eff_ = 45 ± 18 kJ·m^−3^ for MNP-6 compares well with such an enhancement, and a mixture of magnetite and maghemite can explain our measured value for the smallest batch. The observed NP size for blocking at ambient temperature of about 25 nm compares well with the predictions for 25 nm Fe_3_O_4_ cuboids with an aspect ratio of 1.05–1.1 [54]. Such slight elongations are also present in the octahedra.

Overall, the static magnetic properties suggest that the smallest particles are of little interest for MRI and MPH applications. MNP-15 and larger NPs perform better under 100–1000 kHz ac magnetic fields [6,55]. For MRI in quasi-static fields, however, we expect that MNP-25 and MNP-44 will perform the best.

### Magnetic resonance imaging

Knowing the structural and magnetic properties of our hybrid Fe_3_O_4_–Au NPs, in this section we discuss if such features have an impact on the NP performance in MRI. For this purpose, the NPs were stabilized in water by modification with a biocompatible derivative of polyethylene glycol and phospholipid (DSPE-PEG-COOH). The NPs with a polymer shell have a hydrodynamic diameter ranging from 95 to 160 nm, according to the dynamic light scattering data (Table S1, Supporting Information File 1).

The ability of magnetite NPs to increase the *T*_2_-contrast in MRI arises from the creation of huge magnetic field gradients, accelerating the relaxation rate of water protons in the vicinity of the NPs [56]. The correlation of *r*_2_-relaxivity with the size of Fe_3_O_4_ NPs and clusters of NPs has been thoroughly discussed in the literature. See for example [57–60]. These aggregates can be considered as magnetic volumes in which the dipole–dipole interaction between NPs produces a strong magnetic field gradient leading to the predominant *T*_2_-effect. The *r*_2_-relaxivity is affected by NP aggregation, and three different regimes can be distinguished. First, for small clusters, *r*_2_ is given by the theory of the outer sphere. NPs are homogeneously dispersed, and water protons diffuse between the magnetic cores before becoming completely out-of-phase. At this point, *r*_2_ increases with the NP size. This regime is called the motional average regime (MAR). Therefore, MAR is predicted for relatively small iron oxide NPs, where water diffusion near NPs occurs on much faster timescales than the resonance frequency shift, resulting in increased *r*_2_ values with increasing NP size [61]. For example, the variation of NP diameters from 4 nm to 6 nm, 9 nm, and 12 nm resulted in *r*_2_ values of 78, 106, 130, and 218 mM^−1^·s^−1^, respectively [62]. When the diameter is increased further, the *r*_2_ value is constant up to a certain size limit. The size and the corresponding stray field are so large that water molecules experience a nearly constant magnetic field during their *T*_2_-relaxation. These NPs are then in the so-called static dephasing regime (SDR) [63], which determines the relaxivity limit, and the *r*_2_ value reaches a plateau. In the SDR, the induced perturbing field around larger NPs is much stronger, and proton diffusion becomes nondominant for the signal decay. For instance, the *r*_2_ values increased rapidly from 173 to 204 and 240 mM^−1^·s^−1^ at 7 T for NPs from 8 nm to 23 nm and 37 nm, respectively [63]. For larger 65 nm sized iron oxide NPs [64], the *r*_2_-relaxivity only slightly increases further to 249 mM^−1^·s^−1^. Recently, Reguera et al. [65] reported a similar enhancement of Δ*r*_2_ ≈ 100 mM^−1^·s^−1^ for increasing diameters from 16 nm to 20 nm Fe_3_O_4_–Au hybrid NPs. Finally, as the size further increases, *r*_2_ decreases with increasing size. The decrease rate of *r*_2_ depends on the echo time in the partial refocusing model [66].

**Figure 5:**
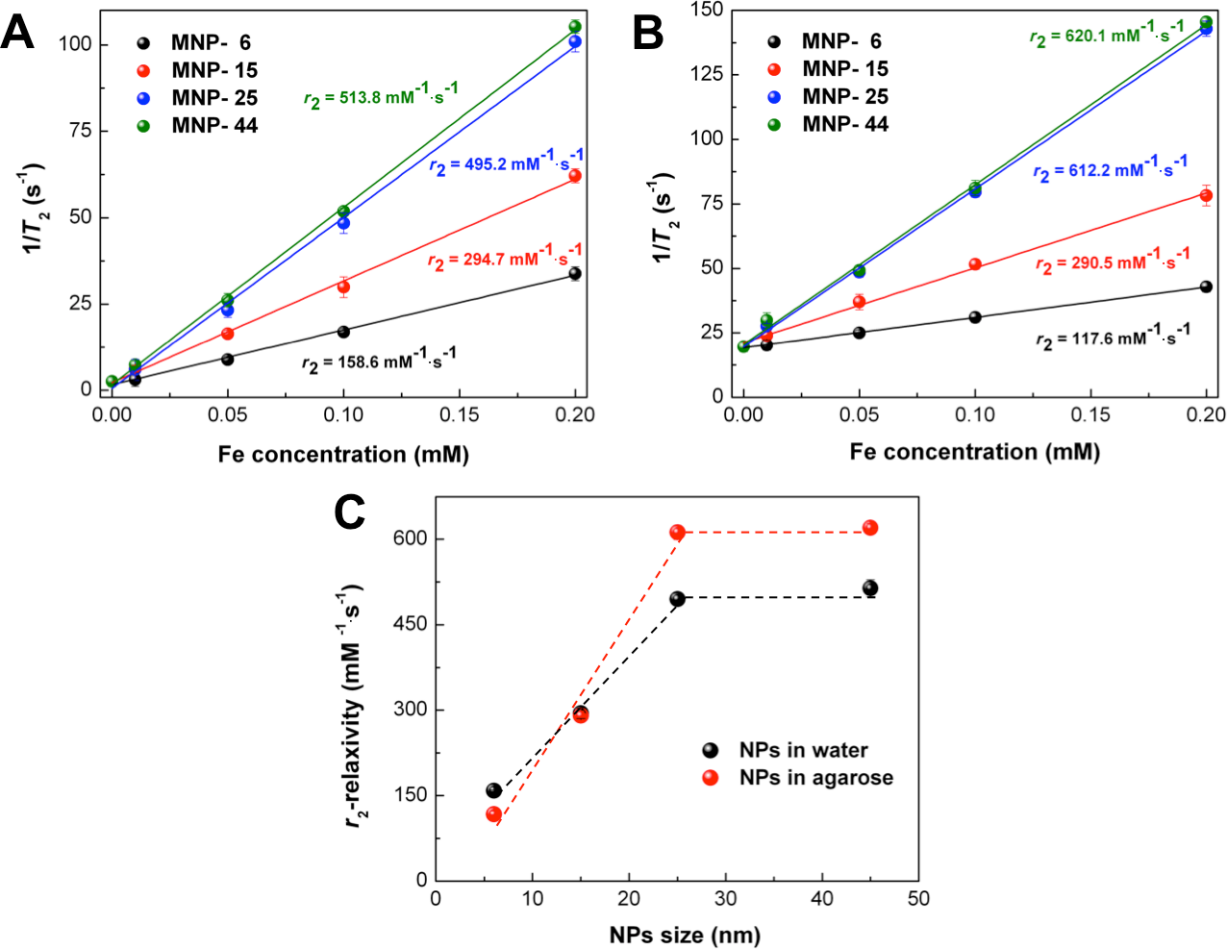
Inverse of the MRI proton *T*_2_-relaxation time as a function of iron concentration for MNP-6, MNP-15, MNP-25 and MNP-44 in water (A) and 2% agarose (B). The *r*_2_ values are determined by the slopes of the linear fits. C) *r*_2_ values as a function of NP size in water and agarose. The SDs are smaller than the symbol size.

In our experiments, the *r*_2_ values of the Fe_3_O_4_–Au hybrid samples were measured for all particle sizes (Figure 5, Figure S3, Supporting Information File 1) in water and in 2% agarose NP solutions. The latter has a viscosity close to that of cell cytoplasm [6,67] thus mimicking the viscosity and microstructure of tissues [68–69]. We measure an increase of the *r*_2_-relaxivity from 159 to 495 mM^−1^·s^−1^ in water (Figure 5A) and from 118 to 612 mM^−1^·s^−1^ in agarose (Figure 5B) for the sample series of MNP-6, MNP-15 and MNP-25 Fe_3_O_4_–Au hybrid samples, while for even larger, 44 nm Fe_3_O_4_–Au NPs (sample MNP-44) no significant increase in *r*_2_ (514 and 620 mM^−1^·s^−1^ for water and agarose solutions, respectively) was observed. We ascribe this to the initial increase of the saturation magnetization *M*_S_ for larger Fe_3_O_4_ NPs from 6 to 25 nm. For MNP-25 the bulk *M*_S_ value is reached and remains roughly constant for MNP-44. The *T*_2_ behavior of samples MNP-6 and MNP-15 can be described as within MAR. Further increase of size (MNP-25) results in an intermediate state close to the SDR regime, while sample MNP-44 is presumably in SDR. It should be mentioned that the *r*_2_-relaxivity of MNP-25 and MNP-44 samples is much higher as compared to other examples of Fe_3_O_4_–Au hybrid NPs (*r*_2_ = 245–381 mM^−1^·s^−1^) [65,70] and commercial contrast agents, such as Feridex^®^, with an *r*_2_ of 120.0 mM^−1^·s^−1^ [71] and Resovist^®^, with an *r*_2_ of 150.0 mM^−1^·s^−1^ [72].

The most probable reasons for the extraordinarily high performance of our NPs are the perfect crystallinity and the resulting bulk-like *M*_S_ leading to a stronger local magnetic stray field [59]. Following Hwang and Freed's theory [73] the *r*_2_ value is proportional to the square of two key parameters in highly magnetized nanomaterials: the *M*_S_ value and the effective magnetic radius *R*. In short, the *M*_S_ value determines the local magnetic field inhomogeneity induced by the NPs. The effective radius is responsible for the field perturbation volumes for water protons. Variations from a spherical shape of the NPs, especially significant for the present octahedra, increase the effective magnetic anisotropy by shape anisotropy which increases quadratically with *M*_S_ [59]. Experimentally, this was observed by Joshi et al. [74] and Smolensky et al. [75] when they compared spherical and faceted NPs. In both studies, a higher *r*_2_-relaxivitiy was found for faceted NPs. These findings also correlate with the highest *r*_2_ values ever reported (761 mM^−1^·s^−1^ for 22 nm cubic Fe_3_O_4_ NPs [76] and 679 mM^−1^·s^−1^ for Fe_3_O_4_ octapods [77]). Therefore, in addition to the stray field strength, the facets of MNP-25 and MNP-44 are likely to produce stronger stray field gradients Δ*B* in their vicinity, especially near the six corners and eight edges of the magnetic octahedrons.

The effective magnetic radius *R* of a nanoplate, for instance, has been determined to be much larger than its spherical counterpart with a similar solid volume [78], which leads to enhanced *r*_2_ values. This principle may be applied to the octahedral particles of MNP-25 and MNP-44 samples as an explanation of increased *r*_2_-relaxivity values. Zhou et al. [78] further argued that the strong *T*_1_ and *T*_2_ contrast enhancement of nanoplates could be explained by the large surface area of Fe_3_O_4_ (111) facets for efficient chemical exchange/interaction. This also holds for the present NPs with (111) facets (see Figure S2C, Supporting Information File 1). Finally, the functionalization of the NPs with DSPE–PEG–COOH further increases the *r*_2_-relaxivity since the subunits of PEG chains are usually associated with two or three water molecules via complex formation and/or hydrogen bonds. These strong interactions slow down the diffusion of water molecules to some extent and increase the *r*_2_ value [59].

Moreover, *r*_2_ values are even higher for the larger NPs dispersed in agarose in comparison with water solutions (Figure 5C), while for the smaller NPs, lower *r*_2_ values are observed. This splits the batches into two size regimes, namely SDR and MAR for the larger and smaller batches, respectively. According to these relaxation regimes, the NPs up to about 20–25 nm are in the MAR where the diffusion processes are the predominant factor for the *r*_2_-relaxivity. This limit correlates well with the model predictions for the *r*_2_ value described in [66] considering clusters of 4–5 NPs in the case of sample MNP-6 (modeled *r*_2_ = 154 mM^−1^·s^−1^) and single NPs of MNP-15 (modeled *r*_2_ = 255 mM^−1^·s^−1^) in MAR. The confinement of the NPs in an agarose matrix hinders or at least slows down the diffusion of water molecules near NPs and therefore decreasing *r*_2_ values are obtained. Although the theoretical limit [66] for MNP-25 and MNP-44 in SDR of ≈1000 mM^−1^·s^−1^ (given their high *M*_S_ values) is not reached, an additional stabilization by agarose seems to play a decisive role for significantly enhanced *r*_2_ values [57]. This means that the effectiveness of the hybrid materials as contrast agents increases under in vitro and in vivo operational conditions.

### Magnetic hyperthermia

**Figure 6:**
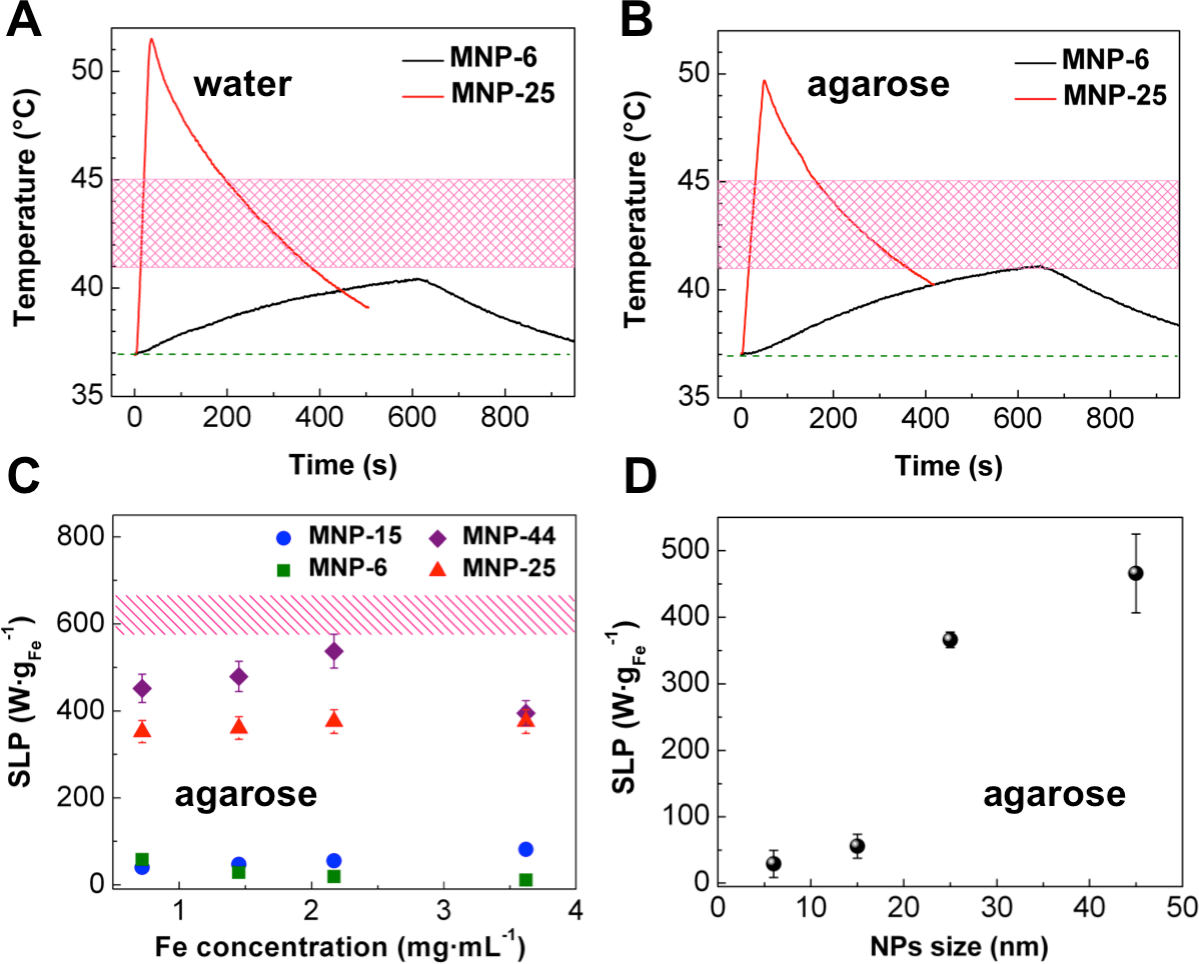
MPH experiments (765 kHz, 30 mT). The heating curves of MNP-6 and MNP-25 (3.6 mg·mL^−1^ Fe) in water (A) and agarose (B), the shaded bands show the 41–45 °C region; SLP values for MNP-6, MNP-15, MNP-25 and MNP-44 samples for various concentrations in agarose compared to the reference values for MNP-25 (3.6 mg·mL^−1^ Fe) in water shown by the shaded band (C); the comparison of concentration-averaged SLP values for the NPs of various size (D). The error bars in (C) and (D) correspond to the standard deviation.

Next, we evaluate the heating efficiency of the hybrid NPs in MPH, measuring the heating rate in both water and agarose at various concentrations in AMF at the frequency of 765 kHz and amplitude µ_0_*H* = 30 mT, as shown in Figure 6. The relatively high AMF frequency has been chosen for better data acquisition. Figure 6A depicts a set of two hyperthermia sequences, composed of a heating (magnetic field is switched on) and a cooling stage (magnetic field is switched off), for two aqueous solutions, respectively. It is apparent that there is a critical magnetite size that renders such structures suitable for hyperthermia applications. Superparamagnetic 6 nm Fe_3_O_4_ are too small to induce a thermal shock within the hyperthermia window of 41–45 °C (shaded temperature band in the figure) while 25 nm Fe_3_O_4_ safely reach 42 °C within the first 35 s of AMF application. A similar, yet moderated situation is shown in the corresponding agarose samples in Figure 6B. Agarose is a polysaccharide matrix, widely accepted as an excellent phantom system since, with respect to its concentration, it may mimic both soft and hard tissues.

From the initial heating rates Δ*T*/Δ*t* we determine the SLP for NPs of a certain size range for agarose medium and MNP-25 in aqueous medium as a reference. Table 4 summarizes the results. The experimental details and evaluations are explained in the Experimental part. For samples MNP-6 and MNP-15, the obtained SLP values are below 80 W·g^−1^, which is insufficient for effective MPH. Reasonably high SLP values, however, are found for MNP-25 and MNP-44 (larger than 327 W·g^−1^). We ascribe the observations to the transition from superparamagnetic to ferrimagnetic behavior between MNP-15 and MNP-25 together with the increasing *M*_S_ with NP size in agreement with literature [28]. In the blocked state, MPH additionally benefits from Néel losses, increasing the SLP. Diameters in the range of 20–25 nm Fe_3_O_4_ are considered to be optimal for iron-oxide-based MPH [79]. Despite this, the observed SLP values for MNP-44 are significantly higher than for MNP-25, which can at least partly be explained by the higher blocking temperature. Thus, all NPs in MNP-44 contribute to the temperature rise via Néel losses. Hysteresis losses prevail and stabilize the SLP values for MNP-25 and MNP-44 at 3.6 mg·mL^−1^ Fe concentration (Figure 6C). As a reference, the heating rate and corresponding SLP are also determined for MNP-25 in aqueous solution (3.6 mg·mL^−1^ Fe), which is used for in vitro hyperthermia experiments in the following. The SLP of 617 ± 44 W·g^−1^ for the MNP-25 sample is indicated by the shaded band in Figure 6C. This value is comparable to the SLP of 524 W·g^−1^ for NP chains in magnetosomes with a 30 nm core size obtained by Hergt et al. [80]. The authors, however, used more moderate field conditions (12.5 mT, 410 kHz) in their experiments.

**Table 4:** The heating rate Δ*T*/Δ*t* and calculated SLP values for various NP concentrations and sizes in agarose and aqueous medium using MNP-25 as a reference.

Sample	Medium	Heating rate (K·s^−1^)	*c*(Fe_3_O_4_) (mg·mL^−1^)	*c*(Fe) (mg·mL^−1^)	SLP (W·g_Fe_^−1^)

MNP-6	agarose	0.010	5	3.6	12 ± 1
MNP-15		0.070			81 ± 6
MNP-25		0.281			327 ± 24
MNP-44		0.342			398 ± 29
MNP-25	water	0.531	5	3.6	617 ± 44

For each sample the SLP values are averaged over all concentrations since we only obtain minor variations with increasing relative amounts. Figure 6D presents the SLP values as a function of NP size. The strongly increasing SLP between MNP-15 and MNP-25 is attributed to the transition from superparamagnetic to the thermally blocked state in this size regime.

Our results are in good agreement with the relevant literature on gold/iron oxide nanoparticle dimers (Fe_3_O_4_–Au [81] and Fe_2_O_3_–Au [82]), where the heating efficiency is optimized at diameters of about 23 nm, and SLP values up to 1330 ± 20 W·g^−1^ (300 kHz, 30 mT) are reported. This high SLP value, however, is questionable, since the heating curve of this sample provided in the supplementary information of [82] for a Fe concentration in the 6–12 mg·mL^−1^ range, only delivers a heating rate of 0.640 K·s^−1^. This corresponds, according to our calculations, to 223–447 W·g^−1^ SLP (depending on the Fe concentrations used). Therefore, we can conclude that our Fe_3_O_4_–Au hybrids with 25 nm diameter provide high SLP values for MPH, which are at least in line with the values reported in [82]. In all cases nonadiabatic correction is performed within the SLP calculation to avoid erroneous overestimations due to heating transfers of nonmagnetic origin [83]. Eventually, a critical magnetite diameter (≥20 nm) is required to promote enhanced heating efficiency within the concentration range of 1–10 mg mL^−1^.

Moreover, the dispersion of NPs in an agarose matrix results in the same SLP magnitudes for MNP-6 when compared to water solutions (see the heating curves, Figure 6A and Figure 6B) and leads to an almost two-fold decrease of SLP for the MNP-25 sample. This decrease is due to Brownian relaxation, which is dependent on the medium viscosity, and is at least partially suppressed in agarose due to the increased hydrodynamic diameter (Figure 6C). The good performance of MNP-25 is very important here since it affirms the application of the NPs for magnetic hyperthermia in conditions comparable to the intracellular environment. Moreover, the SLP values of the larger NPs are adequate to promote significant heating in the in vitro experiments following.

### In vitro test of performance

The high contrast properties of 25 nm Fe_3_O_4_–Au hybrid NPs for in vitro MRI in 4T1 mouse breast adenocarcinoma cells have been recently demonstrated [84]. In summary, a *r*_2_ value of 276.9 mM^−1^·s^−1^ was obtained after 24 h of NP incubation with cell culture. Such an *r*_2_ value is suitable for MRI, although it was found to decrease as compared to the hybrids in water or agarose in line with previous cell culture experiments [85].

**Figure 7:**
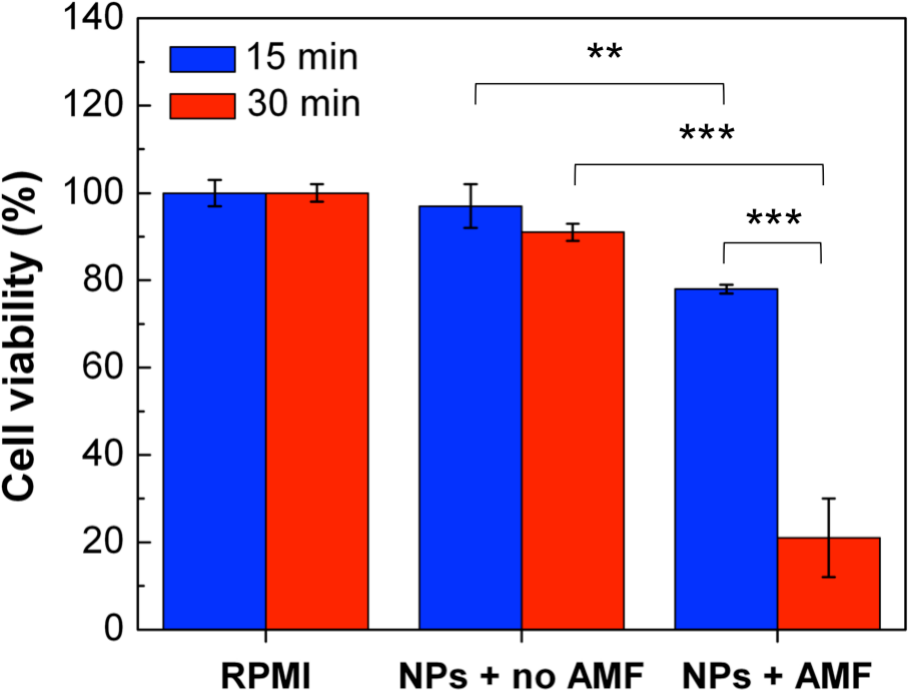
Cell viability study (MTS assay) of 4T1 cells after 15 and 30 min incubation with NPs during AMF exposure or without its application. RPMI: viability of cells cultivated at 37 °C in cell medium without NPs; NPs + no AMF: viability of cells cultivated at 37 °C in the presence of MNP-25 in cell medium for 15 or 30 min; NPs + AMF: viability of cells cultivated in the presence of MNP-25 in cell medium for 15 or 30 min of AMF exposure (heating up to 46 ± 1 °C in 261–393 kHz, 25 mT AMF). The results are shown as the mean ± SD, **p < 0.01, ***p < 0.001 (one-way ANOVA).

Here, we focus on the hyperthermia function of the hybrid materials in the same cell line. For this purpose, polymer-coated MNP-25 NPs were dispersed in RPMI medium at 3.6 mg·mL^−1^ Fe concentration, resulting in the same hydrodynamic size as in water (Table S1, Supporting Information File 1) and added to 4T1 cells. The specimen was immediately exposed to 261–393 kHz, 25 mT AMF. The frequency is adjusted to keep the temperature constant at 46 ± 1 °C for 15 or 30 min. Afterwards, the cell viability is tested by several methods. Standard MTS assay (Figure 7, Table S2, Supporting Information File 1) was conducted to investigate the NP cytotoxicity. These results are supplemented with apoptosis/necrosis activation (Figures S4 and S6, Supporting Information File 1) and production of reactive oxygen species (ROS) (Figures S5 and S7, Supporting Information File 1). The ROS excess level is known to induce apoptosis [86–88]. The applied combination of techniques enables us to draw definite conclusions about the effect of NPs on cell viability [89].

In our experiments, 15 min AMF exposure of 4T1 cells incubated with NPs indicate the initial level of induced cell death (22 ± 1%, MTS assay) in comparison with cells, incubated at 37 °C with NPs in the absence of AMF and control samples without NPs in zero field or exposed to AMF. This is well in line with the detection of apoptosis/necrosis as a positive staining of 4T1 cells is found only on the periphery of the cell monolayer. However, ROS activation is observed at this point of time – as indicated by an increased number of H2DCFDA-positive cells in the culture (Figure S5C, Supporting Information File 1). Exposure to AMF for 30 min is sufficient to kill 79 ± 8% of cells according to the MTS assay. Consistent with this finding, more pronounced apoptosis/necrosis activation is detected (Figure S5D, Supporting Information File 1).

Next, 4T1 cells are precultivated with NPs for 6 h before AMF exposure to increase NP–cell interactions. In this case, 15 min and 30 min of exposure to AMF led to similar, yet improved, results: 100% cell death detected by apoptosis/necrosis activation in cell culture (Figure S6, Supporting Information File 1). Accordingly, no ROS activation is detected (Figure S7C and S7D, Supporting Information File 1) due to the late stage of apoptosis.

The two sets of experiments, direct AMF treatments and precultivation for 6 h, both show clear apoptosis/necrosis of 4T1 cells induced by the hyperthermia treatment. The more efficient precultivation might help to decrease the concentration threshold for MPH in future studies. Furthermore, the results are in line with previous reports on polymer-coated Fe_3_O_4_ or MnFe_2_O_4_ NPs, where apoptosis/necrosis of various cell cultures along with ROS generation was observed [90–94] for the identical concentration range (1–10 mg·mL^−1^) and similar AMF conditions. Other experiments have shown a decrease of 4T1 cell viability down to 60–70% after initial incubation of cells with magnetic NPs [95–96]. In the current study, however, we achieve the same level of cell viability immediately after NP addition followed by AMF treatment. If the cells are first incubated with NPs for 6 h before field application, 100% cell death is observed. Therefore, the results demonstrate not only the general in vitro function of Fe_3_O_4_–Au hybrid NPs for MPH, but also the opportunity of reduced AMF treatment time leading to 100% cell death if an intermediate step of NP–cell co-cultivation is added. Corato et al. [81] have tested Fe_3_O_4_–Au NPs for hyperthermia treatment of so-called “minitumors”, consisting of SKOV-3 cells, which is the transitional step between in vitro and in vivo experiments. Considering the higher SLP values of the present hybrid NPs, the suggested approach may improve in vivo MPH in future experiments.

## Conclusion

We have successfully synthesized Fe_3_O_4_–Au hybrid NPs with 6–44 nm diameter Fe_3_O_4_ and 3–11 nm diameter Au subunits, while maintaining an approximately constant Fe_3_O_4_/Au volume ratio. With the increase of size, the iron oxide lattice parameters change towards stoichiometric Fe_3_O_4_. Hybrids below 20 nm are superparamagnetic, while NPs of larger diameter are thermodynamically blocked, and the Verwey transition is observed in ZFC/FC curves as an indicator of high quality, bulk-like Fe_3_O_4_. The best combination of the *r*_2_-relaxivity and SLP values for all samples, both in water and agarose mimicking tissues, is obtained for 25 nm diameter Fe_3_O_4_–Au NPs. This also allows for efficient NP visualization and heating in in vitro conditions, leading to the death of 4T1 mouse breast adenocarcinoma cells in high-frequency alternating magnetic fields. Therefore, these hybrid nanomaterials are demonstrated to exhibit an optimized theranostic response in magnetic resonance imaging and magnetic particle hyperthermia.

## Experimental

### Materials

Iron pentacarbonyl Fe(CO)_5_, hydrogen tetrachloroaurate trihydrate (III) HAuCl_4_∙3H_2_O, oleic acid, oleylamine, phenyl ether, benzyl ether, 1-octadecene and 1,2-hexadecanediol were purchased from Sigma-Aldrich. 1,2-Distearoyl-*sn*-glycero-3-phosphoethanolamine-*N*-[carboxy(polyethylene glycol)-5000] ammonium salt (DSPE-PEG-COOH) was delivered by Avanti Polar Lipids. Isopropanol, hexane and chloroform were purchased from Reachim. Water used in the experiments was deionized (18.2 MΩ·cm^−1^, Millipore Milli-Q Academic System).

### Synthesis and functionalization of nanoparticles

The Au NPs were synthesized according to a previously published protocol [97]. Briefly, 35 mg HAuCl_4_∙3H_2_O was dissolved in 80 mL deionized water (DI H_2_O) and heated up to 80 °C. Then 200 μL oleylamine was added and the temperature was maintained during 3 h. After cooling down to room temperature, the water was evaporated, and the Au NPs were redispersed in hexane (2 mL).

The synthesis of MNP-6 and MNP-15 samples was performed as follows: Fe_3_O_4_–Au hybrid NPs with in situ synthesized Au seeds were obtained by thermal decomposition of Fe(CO)_5_ and HAuCl_4_∙3H_2_O at high temperatures following a modified protocol [98]. In brief, a mixture of 20 mL high-boiling solvent (phenyl ether for sample MNP-6 or 1-octadecene for sample MNP-15), 2.584 g 1,2-hexadecanediol, 2 mL oleylamine and 2 mL oleic acid was heated up to 120 °C under argon atmosphere and kept at this temperature for 30 min. Then, 0.28 mL of Fe(CO)_5_ was added. Three minutes later, a mixture of HAuCl_4_∙3H_2_O (45 mg), 5 mL solvent and 0.5 mL oleylamine was added, and the final solution was slowly (3 °C/min) heated up to reflux for 45 min. After cooling down to room temperature, the reaction mixture was oxidized by stirring for 1 h under ambient air. The NPs were isolated via centrifugation, washed several times with isopropanol and dispersed in hexane.

Fe_3_O_4_–Au hybrid NPs with presynthesized Au seeds (MNP-25 and MNP-44) were grown by thermal decomposition of Fe(CO)_5_ in the presence of Au NPs following a modified protocol [84,99]. A mixture of 1 mL oleic acid and 20 mL solvent (phenyl ether for sample MNP-25 or benzyl ether for sample MNP-44) was heated up to 120 °C under argon atmosphere and kept at this temperature for 30 min. Then, 0.28 mL of Fe(CO)_5_ was added. Five minutes later, the presynthesized Au NPs in 2 mL hexane and 500 μL oleylamine were added, and the final solution was slowly heated up to reflux at a rate of 3 °C/min for a total time of 3 h. After cooling down to room temperature, the reactants were oxidized by stirring for 1 h under ambient air. The NPs were isolated via centrifugation, washed with isopropanol and dispersed in hexane.

Oleic-acid-coated Fe_3_O_4_–Au NPs were transferred into water by modification with 1,2-distearoyl-*sn*-glycero-3-phosphoethanolamine-*N*-[carboxy(polyethylene glycol)-5000] ammonium salt (DSPE–PEG–COOH) [100]. Fe_3_O_4_–Au NPs (1 mg) and DSPE–PEG–COOH (2.45 mg) were mixed in 1 mL chloroform via ultrasonication. The mixture was left overnight for the slow evaporation of the solvent. Then DI H_2_O was added to the precipitate, and the solution was sonicated for 15 min. After that, unbound polymer was removed by centrifugation (14500 rpm for 30 min) twice. Finally, the NPs were redispersed in 2 mL DI H_2_O.

### Characterization techniques

All particle batches were examined by a JEOL JEM-1400 transmission electron microscope operated at 120 kV acceleration voltage. Overview images were taken in conventional bright-field TEM mode. The samples were prepared by casting and evaporating a droplet of hexane solution onto a carbon-coated copper grid (300 mesh). The average diameter of NPs was calculated from TEM images by analysis of about 500 NPs for each sample using ImageJ software. Selected samples were investigated in bright-field high-resolution mode using a FEI Tecnai F20 microscope operated at 200 kV acceleration voltage. The Fe and Au concentrations in the samples were measured by microwave-coupled plasma atomic emission spectrometry (Agilent 4200 MP-AES, USA) for the NPs dissolved in aqua regia using the calibration curve for the standard samples in 0.1–1 mg·mL^−1^ concentration range.

X-ray diffraction patterns were measured from 2θ = 30° to 120° at a scan rate of 0.1° per step and 3 s per point using the X-ray powder diffractometer Rigaku Ultima IV with Co Kα radiation and a graphite monochromator on the diffracted beam. Quantitative XRD analysis (including crystal size evaluation by determination of the coherent scattering region) was performed using the PHAN% and SPECTRUM programs developed by the Physical Materials Science Department of the National University of Science & Technology (NUST) “MISIS” that are a modification of the Rietveld method, based on the minimization of the difference between the experimental spectrum, taken from the points, and model (calculated) one. For fitting the spectra, the lattice parameters, the amount of each phase and their crystallite diameter are optimized.

Standard magnetometry at various temperatures and fields was measured in a Quantum Design PPMS DynaCool system. For this, about 10 mg of dried powder Fe_3_O_4_–Au NPs was put into synthetic capsules.

The hydrodynamic size of the NPs in water was measured by dynamic light scattering using a Zetasizer Nano ZS (Malvern Instruments). The average values with error bars were obtained from three measurements of each sample.

Magnetic resonance imaging (MRI) was measured at 18 °С in a ClinScan 7 T MRI system. The *r*_2_-relaxivity of hydrogen protons in the presence of Fe_3_O_4_–Au NPs modified with DSPE–PEG–COOH was determined by linear fitting of various Fe concentrations from 0.01 to 0.2 mM in water and 2% w/w agarose. Image acquisition was performed in the spin echo mode with the following parameters: repetition time 10 s, echo times 16, 24, …, 256 ms, flip angle 180°, resolution 640 × 448 pixel, field of view 120 × 82.5 mm^2^. The signal intensities were determined using ImageJ software, and the *T*_2_-relaxation time was calculated by exponential fitting as a function of echo time. The *r*_2_-relaxivity values were calculated from linear fitting of *T*_2_^−1^ relaxation times as a function of Fe concentration.

Magnetic particle hyperthermia (MPH) experiments were performed using a commercial 4.5 kW inductive heater operating at 765 kHz under AC induction amplitudes of up to 30 mT. Each measurement cycle included a heating and a cooling stage. The temperature was continuously recorded (0.4 s steps) by a GaAs-based fiber optic probe immersed in the vial containing 1 mL of solution. The heating efficiency of the NPs is quantified by the specific loss power (SLP) determined from the power absorption per unit mass of magnetic material (in W·g_Fe_^−1^) following a standardized procedure to estimate solely the magnetic heating contribution by using


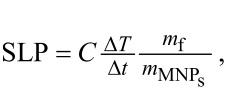


where *C* is the volumetric specific heat capacity of the sample, *m*_f_ the dispersion mass, *m*_MNPs_ is the iron mass diluted in the dispersion and Δ*Τ*/Δ*t* is the maximal slope at initial time after switching on the heating AC field.

### In vitro experiments

**Cell culture.** 4T1 mouse breast cancer cells were purchased from the American Type Culture Collection (ATCC, Manassas, VA, USA). They were cultured in RPMI-1640 medium (Gibco) supplemented with 10% fetal bovine serum (FBS) (Gibco) and 2 mM L-glutamine (Gibco) at 37 °C in a humidified incubator supplied with 5% CO_2_.

**MTS assay.** The cells were plated in the wells of Stripwell 96-well plates (Corning) at a concentration of 6,000 cells per well. The cells were counted using the automatic cell counter EVE. After two days the medium from the cells was replaced by 200 μL of the Fe_3_O_4_–Au Janus NP solution in full culture medium (the final concentration of NPs was 3.6 mg∙mL^−1^ Fe), and the obtained samples were exposed to high-frequency AMF (TOR Ultra HT, Nanomaterials LLC, Russia) for 15 min or 30 min immediately or 6 h after NP–cell co-cultivation. The AMF parameters of 261–393 kHz, 25 mT were used to keep a constant temperature of 46 ± 1 °C (checked by Seek Thermal camera and software). The cells, incubated in the full culture medium and in a medium with the same concentration of NPs at 37 °C without AMF, were used as controls. After hyperthermia treatment, the medium with NPs was replaced by 100 μL of new culture medium, and 20 μL of MTS reagent (CellTiter 96 aqueous non-radioactive cell proliferation assay, Promega, USA) was added to each well. Following 4 h of incubation at 37 °C in darkness, the wells were placed on a permanent magnet to remove the NPs from solution, and 100 μL of the obtained solution was carefully replaced in the new 96-well plate. The absorbance of the solution was measured at 490 nm using a Thermo Scientific Multiskan GO spectrometer.

**Apoptosis/necrosis detection.** In parallel with the preparation of samples for MTS assay we prepared samples for cell death detection using an apoptosis/necrosis detection kit (abcam). Apopxin deep red dye stained phosphatidylserine on the membrane of apoptotic cells and nuclear green dye – the cells with loss of plasma membrane integrity (i.e., cells at late stage apoptosis or necrotic cells). After the hyperthermia treatment, the cells were washed twice with HBSS (Gibco) supplemented with 2 mM L-glutamine (Gibco) and 10 mM HEPES (Helicon, pH 7.4 adjusted with 1 M NaOH), and intravitally stained with the apoptosis/necrosis detection kit for 40 min at room temperature in the darkness, and washed with full HBSS two times again. The cells, incubated in full culture medium and in medium with NPs at 37 °C without AMF, were used as controls. The obtained preparations were analyzed using a fluorescence microscope (EVOS, life technologies), with a PlanFluor objective 10×/0.3. The further processing of the photos was carried out by ImageJ software.

**ROS detection by 2',7'-dichlorodihydrofluorescein diacetate (H2DCFDA).** Reactive oxygen species (ROS) generation by cells was also investigated during hyperthermia in vitro experiments. In this case, unfixed cells (exposed to AMF and control cells) were washed twice with HBSS supplemented with 2 mM L-glutamine and 10 mM HEPES (pH 7.4 adjusted with 1 N NaOH), and stained with 2 µM H2DCFDA solution (life technologies) for 30 min at 37 °C in darkness. Then the cells were carefully washed with HBSS three times for 5 min. The obtained preparations were analyzed using the EVOS fluorescence microscope with a PlanFluor objective 10×/0.3. The further processing of the photos was also carried out by ImageJ software.

**Statistical analysis.** All data were obtained in three independent triplicate experiments. The percentage of live cells in the MTS assay was represented as the mean ± standard deviation (SD) (for 3 repetitions in each experiment). Plotting and calculation of the standard deviation values were made using Origin 8.0 software. The *p*-values were calculated using one-way ANOVA calculator. *p*-values <0.05 were considered significant (** for *p* < 0.01, *** for *p* < 0.001). A post-hoc Scheffe test was applied.

## Supporting Information

Size distribution for all synthesized NPs (Figure S1), HRTEM images for MNP-6, MNP-44 and MNP-25 samples (Figure S2), *T*_2_-weighted MRI-images of the NP solutions in water and 2% agarose (Figure S3), hydrodynamic size of NPs in water (Table S1), a cell viability study by MTS assay (Table S2), apoptosis/necrosis activation (Figures S4 and S6) as well as reactive oxygen species generation (Figures S5 and S7) in 4T1 cells cultivated with MNP-25 NPs followed by AMF application in comparison with control, are presented in the Supporting Information.

File 1 Additional experimental information.
