# Supplementary material for: Size-selected Fe3O4–Au hybrid nanoparticles for improved magnetism-based theranostics
Source: Beilstein J Nanotechnol. 2018 Oct 16;9:2684–99. doi: 10.3762/bjnano.9.251 (PMC6204820; doi:10.3762/bjnano.9.251)
Supplement: File 1 — Additional experimental information. [file Beilstein_J_Nanotechnol-09-2684-s001.pdf]

# Supporting Information

**for**

# Size-selected $Fe_3O_4$–Au hybrid nanomaterials for improved magnetism-based theranostics

Maria V. Efremova[1,2,‡], Yulia A. Nalench[2,‡], Eirini Myrovali[3], Anastasiia S. Garanina[1,2], Ivan S. Grebennikov[2], Polina K. Gifer[2], Maxim A. Abakumov[2,4], Marina Spasova[5], Makis Angelakeris[3], Alexander G. Savchenko[2], Michael Farle[5], Natalia L. Klyachko[1,2], Alexander G. Majouga[1,2,6] and Ulf Wiedwald*[2,5]

Address: [1]Department of Chemistry, Lomonosov Moscow State University, Moscow, 119991, Russia, [2]National University of Science and Technology «MISIS», Moscow, 119049, Russia, [3]Physics Department, Aristotle University of Thessaloniki, Thessaloniki, 54124, Greece, [4]Department of Medical Nanobiotechnology, Russian National Research Medical University, Moscow, 117997, Russia, [5]Faculty of Physics and Center for Nanointegration Duisburg-Essen, University of Duisburg-Essen (CENIDE), Duisburg, 47057, Germany and [6]D. Mendeleev University of Chemical Technology of Russia, Moscow, 125047, Russia

Email: Ulf Wiedwald - ulf.wiedwald@uni-due.de

* Corresponding author

‡ These authors contributed equally

## Additional experimental information

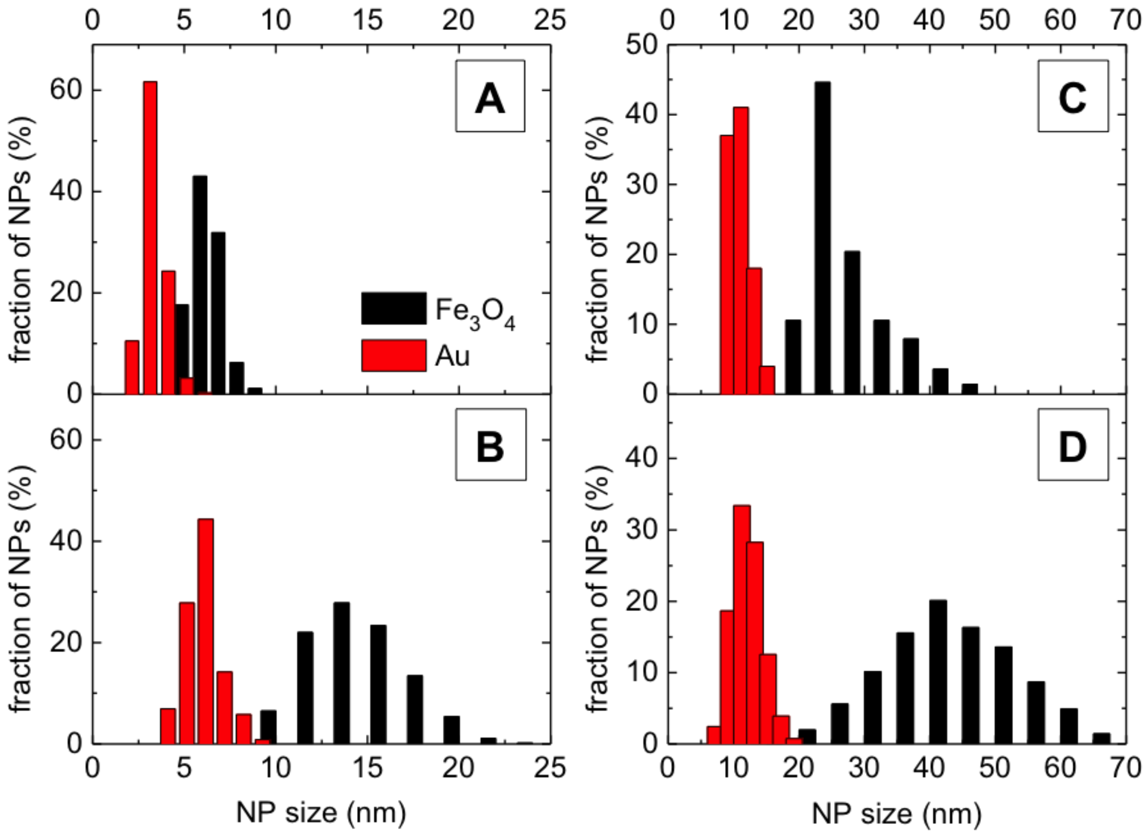


**Figure S1:** Size distributions calculated from TEM images of magnetite (black) and gold (red) parts of NPs for the samples with in situ synthesized Au seeds: A) MNP-6; B) MNP-15; and with pre-synthesized Au seeds: C) MNP-25; D) MNP-44.

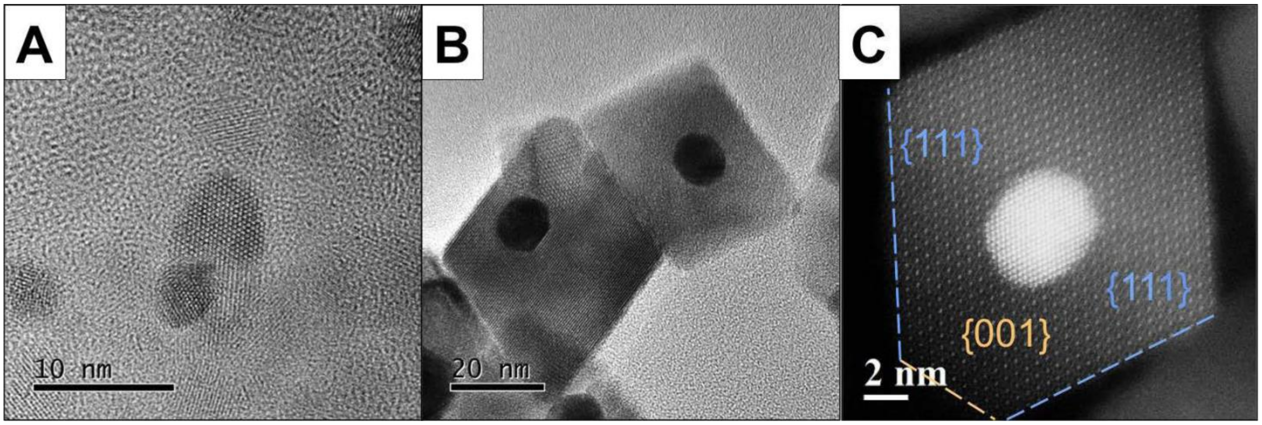


**Figure S2:** HRTEM images of size-selected magnetite-gold NPs: MNP-6 (A), MNP-44 (B), MNP-25 (C).

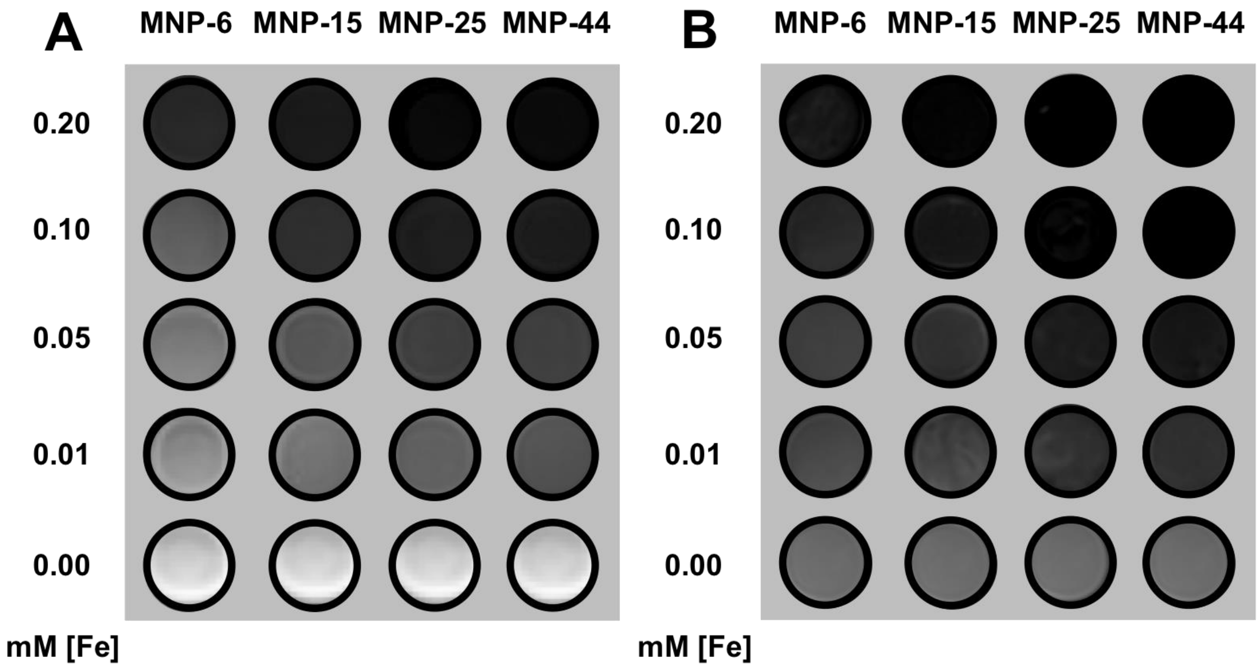


**Figure S3:** Series of $T_2$-weighted images of NPs solutions in water (A) and 2% agarose (B) acquired at TE = 48 ms and concentrations ranging from 0.00 to 0.20 mM Fe.

**Table S1:** Hydrodynamic size of hybrid $Fe_3O_4$-Au NPs, stabilized in water by DSPE-PEG-COOH determined by dynamic light scattering. Results are shown as means ± standard deviation.

| Sample | Hydrodynamic diameter, nm |
|---|---|
| MNP-6 | 95 ± 2 |
| MNP-15 | 112 ± 3 |
| MNP-25 | 121 ± 5 |
| MNP-44 | 160 ± 9 |
| MNP-25 in RPMI | 123 ± 7 |

**Table S2:** A cell viability study (MTS assay) of 4T1 cells after 15 min and 30 min of incubation: "RPMI" – culture medium without NPs at 37 °C; "NPs + no AMF" – in the presence of MNP-25 in cell medium at 37 °C; "NPs + AMF" – in the presence of MNP-25 in the cell medium heated up to 46 ± 1 °C in 261–393 kHz, 25 mT AMF.

| Sample | 15 min | 30 min |
|---|---|---|
| RPMI | 100 ± 3 | 100 ± 2 |
| NPs + no AMF | 97 ± 5 | 91 ± 2 |
| NPs + AMF | 78 ± 1 | 21 ± 9 |

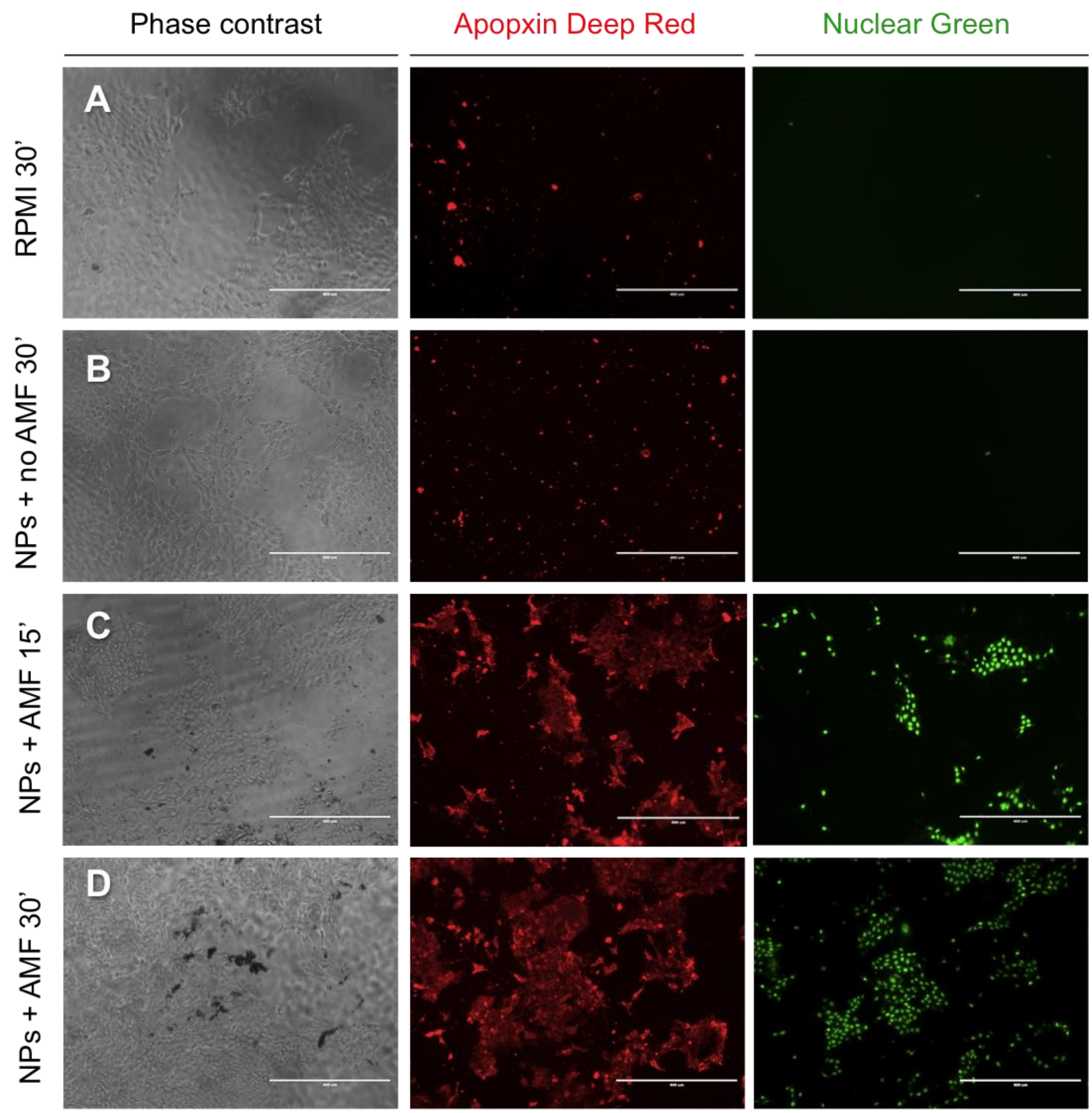


**Figure S4:** Apoptosis/necrosis activation in 4T1 cells, cultivated with polymer-stabilized MNP-25 NPs (3.6 $mg \cdot mL^{-1}$ Fe), phase contrast (left column) and fluorescent microscopy, intravital staining with Apopxin Deep Red (middle column) and Nuclear Green (right column). A) Control cells without NPs; B) cells incubated with NPs at 37 °C in zero AMF; C) cells incubated with NPs in 261–393 kHz, 25 mT AMF (46 ± 1 °C) during 15 min; D) cells incubated with NPs in 261–393 kHz, 25 mT AMF (46 ± 1 °C) during 30 min. Scale bar corresponds to 400 µm.

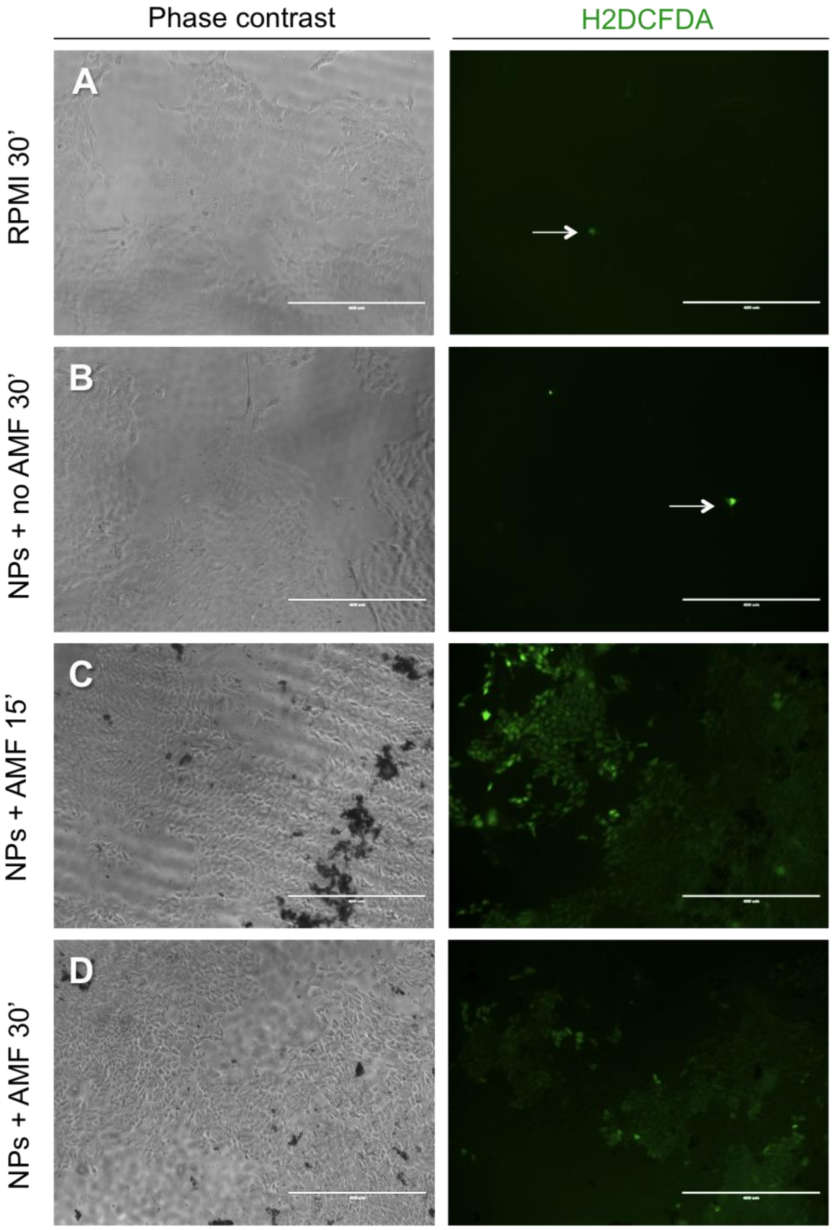


**Figure S5:** Reactive oxygen species (ROS) production by 4T1 cells, cultivated with polymer-stabilized MNP-25 NPs (3.6 mg·mL$^{-1}$ Fe), phase contrast (left column) and fluorescent microscopy, intravital staining with H2DCFDA (right column). A) Control cells without NPs; B) cells incubated with NPs at 37 °C in zero AMF; C) cells incubated with NPs in 261–393 kHz, 25 mT AMF (46 ± 1 °C) during 15 min; D) cells incubated with NPs in 261–393 kHz, 25 mT AMF (46 ± 1 °C) during 30 min. White arrows show single cells with increased level of ROS production. Scale bar corresponds to 400 µm.

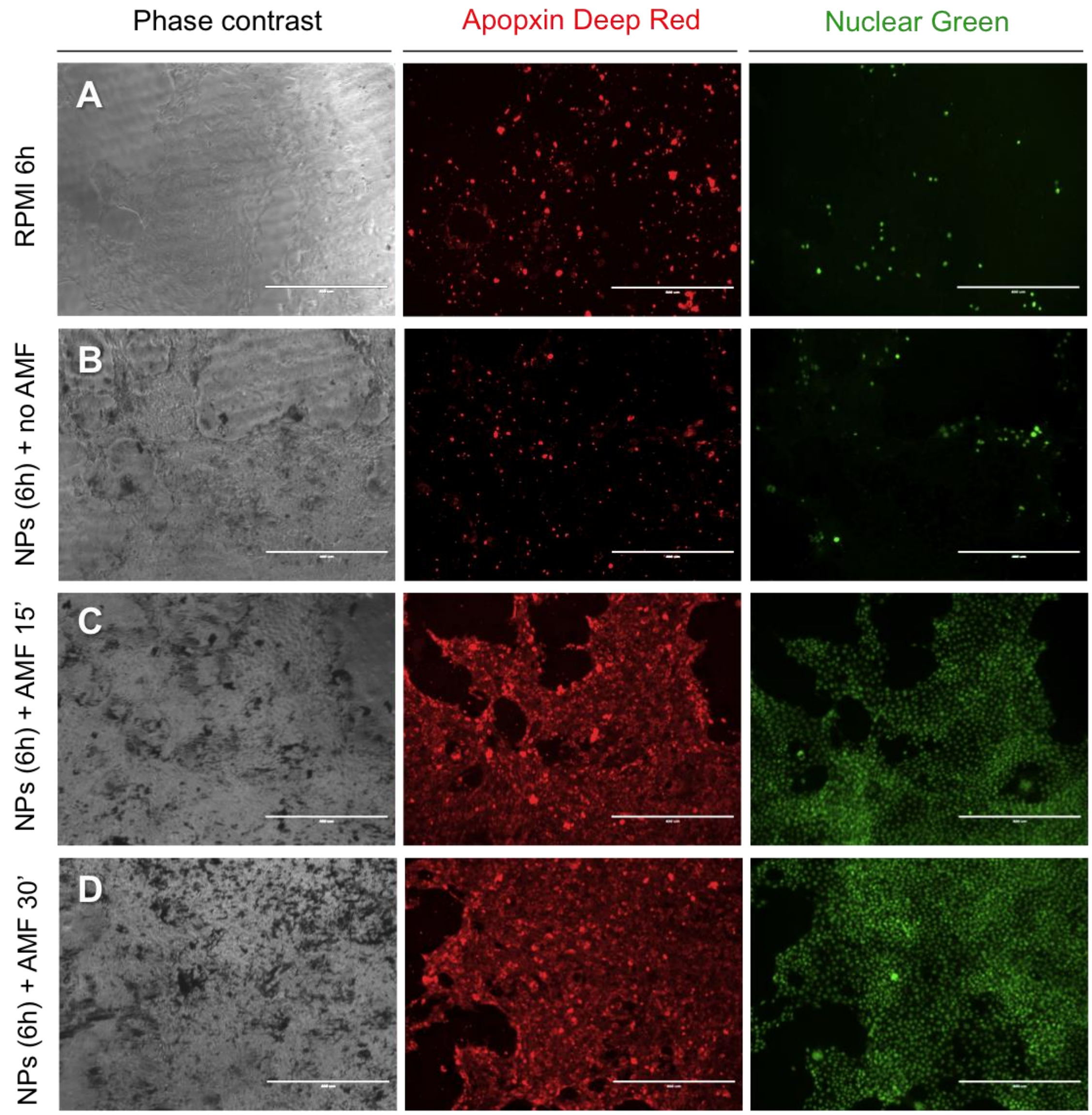


**Figure S6**: Apoptosis/necrosis activation in 4T1 cells, preliminary cultivated with polymer-stabilized MNP-25 NPs (3.6 mg·mL$^{-1}$ Fe) during 6 h, phase contrast (left column) and fluorescent microscopy, intravital staining with Apopxin Deep Red (middle column) and Nuclear Green (right column). A) Control cells without NPs; B) cells incubated with NPs at 37 °C in zero AMF; C) cells incubated with NPs in 261–393 kHz, 25 mT AMF (46 ± 1 °C) during 15 min; D) cells incubated with NPs in 261–393 kHz, 25 mT AMF (46 ± 1 °C) during 30 min. Scale bar corresponds to 400 µm.

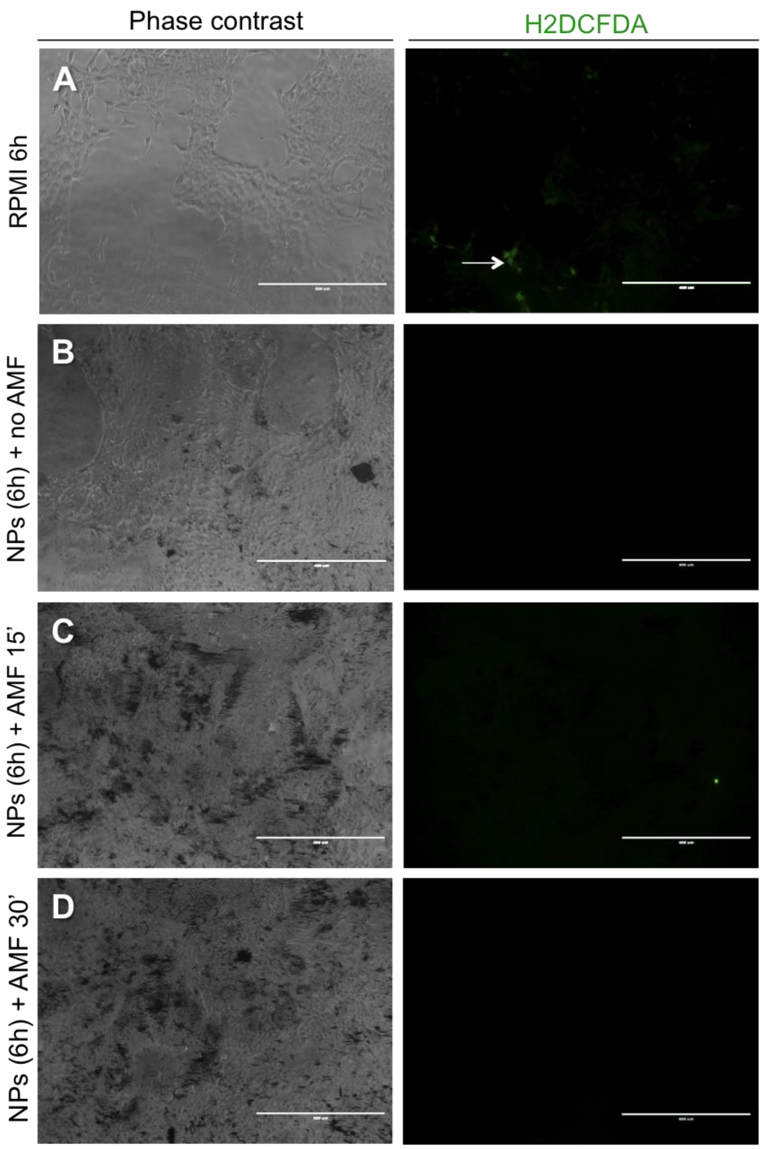


**Figure S7:** Reactive oxygen species (ROS) production by 4T1 cells, preliminary cultivated with polymer-stabilized MNP-25 NPs (3.6 mg·mL$^{-1}$ Fe) during 6 h, phase contrast (left column) and fluorescent microscopy, intravital staining with H2DCFDA (right column). A) Control cells without NPs; B) cells incubated with NPs at 37 °C in zero AMF; C) cells incubated with NPs in 261–393 kHz, 25 mT AMF (46 ± 1 °C) during 15 min; D) cells incubated with NPs in 261–393 kHz, 25 mT AMF (46 ± 1 °C) during 30 min. White arrows show single cells with increased level of ROS production. Scale bar corresponds to 400 μm.
